# Rare Primary Headaches in Children: A Narrative Review

**DOI:** 10.3390/biomedicines14020291

**Published:** 2026-01-28

**Authors:** Edvige Correnti, Sofia D’Agostino, Federica Cernigliaro, Floriana Ferro, Giulia Manfrè, Caterina Gaspari, Carola Meo, Mariarita Capizzi, Giuseppe Giglia, Vittorio Sciruicchio, Vincenzo Raieli

**Affiliations:** 1 Child Neuropsychiatry Department, ISMEP, ARNAS Civico, 90127 Palermo, Italy; edvige.correnti@arnascivico.it (E.C.); fede.cernigliaro@gmail.com (F.C.);; 2 Child Neuropsychiatry Unit Department, Pro.MI.S.E. “G. D’Alessandro”, University of Palermo, 90127 Palermo, Italy; 3 Department of Biomedicine, Neuroscience and Advanced Diagnostics (BIND), Section of Human Physiology, University of Palermo, 90134 Palermo, Italy; giuseppe.giglia@unipa.it; 4 Children Epilepsy and EEG Center, San Paolo Hospital, ASL Bari, 70132 Bari, Italy

**Keywords:** headaches, children, pediatric case, migraine, international headache classification, orofacial pain, red ear syndrome

## Abstract

Headache is a very common disorder in children and adolescents. While migraine and tension headaches are well-known and diagnosed by pediatricians, a group of primary headaches in children, rare in frequency, are poorly understood and likely underestimated by physicians, resulting in delayed diagnosis and treatment. This review aims to provide an updated overview of these clinical forms, considering new evidence. We will present the main clinical, therapeutic, and pathophysiological aspects and possible future hypotheses, with specific reference to pediatric cases of the following clinical forms: cough headache, thunderclap headache, cold headache, primary stabbing headache, nummular headache, hypnic headache, red ear syndrome, and non-odontogenic orofacial pain. These clinical forms currently pose a major diagnostic challenge for pediatricians and represent a source of serious disability for children and adolescents.

## 1. Introduction

According to the third edition of the International Classification of Headache Disorders (ICHD-3) [1], among the four major groups of primary headaches there are some extremely rare cephalalgic syndromes. Despite the low prevalence of these conditions, it is crucial to recognize and differentiate them from more common headaches to exclude secondary causes and choose therapeutic strategies tailored on the specific type of headache.

Pediatric population is prone to show unusual and rare headache syndromes, which are often unknown and misdiagnosed. Within this selection, particularly significant examples include, in order of classification: cough headache, thunderclap headache, cold headache, primary stabbing headache, nummular headache, and hypnic headache [1]. In addition, we describe further interesting and exceedingly rare clinical entities, such as Red Ear Syndrome [2] and non-odontogenic orofacial pain [3], which suggest intriguing nosological, diagnostic, and therapeutic questions.

In this review, we chose not to include the following headaches from the fourth group: primary exertional, sexual, and compression headaches [1], as we did not find any pediatric case studies that would have required a description based solely on data referring to the adult population. New daily persistent headache [1] was not included because it is not rare and, above all, because the complex clinical phenotype, the controversial pathophysiology, and the therapeutic issues required more space than our review could allow.

This review aims to outline the main features of these conditions, with particular emphasis on the developmental age, focusing on their distinctive clinical presentations and the therapeutic options currently available. The goal is to provide significant support for clinicians in order to mitigate the potential repercussions of a missed diagnosis on the quality of life of affected individuals, and to serve as a comprehensive reference for an approach to rare nosological entities in the pediatric and adolescent population.

### Methods

We performed searches in PubMed and Google Scholar for the keywords: “Primary Cough Headache”; “Primary Cough Headache and Children”; “Thunderclap headache”; “Thunderclap headache and Children”; “Cold-stimulus Headache”; “Cold- stimulus headache and Children”; “Primary Stabbing Headache”; “Primar Stabbing Headache and Children”; “Nummular headache”; “Nummular Headaches and Children”; “Hypinc headache”, “Hypnic headache and Children”; “Red ear syndrome”; “Red Ear Sindrome and Children”; “Facial Pain”, “Orofacial Pain”; “Facial Pain and children”; “Orofacial Pain and Children”.

## 2. Primary Cough Headache

Primary cough headache (PCH) is defined by the International Classification of Headache Disorders (ICHD-3) [1] as an acute head pain triggered by Valsalva maneuvers such as coughing, sneezing, or straining for defecation, in the absence of underlying intracranial pathology (see Table 1). While it is well-documented in the adult population, its incidence in pediatric patients is extremely low and potentially underdiagnosed.

### 2.1. Epidemiology and Reported Cases

PCH accounts for about 1% of headaches evaluated in neurological settings [4]. In pediatric age, we only report the case of a 7-year-old boy with a headache triggered by pertussis and associated with phlebectasia of the internal jugular vein [5]. In this case, the headache was intense, daily, lasting about 30 min, and unresponsive to common symptomatic medications. Cervical ultrasound during a Valsalva maneuver showed significant dilation of the right internal jugular vein. 

### 2.2. Pathophysiology

The pathophysiology of PCH is not fully elucidated. In adults, a correlation has been hypothesized with insufficiency of the internal jugular vein valve and the transient increase in intracranial pressure during coughing or other Valsalva maneuvers [6,7]. This mechanism may lead to cerebral venous reflux and activation of the trigeminal nerve, causing pain [8,9]. In children, however, the role of these venous alterations has not been fully confirmed, although some morphological abnormalities, such as jugular phlebectasia, may increase susceptibility to the disorder [10].

### 2.3. Differential Diagnosis

Because 40% of cough headaches are secondary, it is essential to exclude structural malformations such as Chiari type I malformation using brain MRI [4]. The differential diagnosis also includes migraine, trigeminal autonomic cephalalgias, and symptomatic forms due to increased intracranial pressure.

### 2.4. Treatment

In most adult cases, PCH responds to indomethacin [4]. However, in childhood, the approach is more cautious. In the case reported by Omata et al. [5], the headache resolved spontaneously with remission of pertussis, without the need for prophylactic therapy. Identification of an underlying venous abnormality can guide management and reduce family anxiety.

### 2.5. Conclusions

Cough headaches are rare in childhood but can represent a clinically significant condition. Early diagnosis, exclusion of secondary pathologies, and evaluation of venous abnormalities can facilitate targeted and reassuring management. Inclusion of PCH in the differential diagnosis of acute pediatric headaches should be considered, especially in the presence of an evident tussive trigger.

## 3. Thunderclap Headache

According to the criteria of the International Classification of Headache Disorders (ICHD-3) [1], thunderclap headache is included among primary headaches. The diagnostic criteria are the presence of severe headaches with abrupt onset that reach maximum intensity in <1 min, lasting at least 5 min, and not better accounted for by another ICHD-3 diagnosis (see Table 2). In adults [11], thunderclap headache is often associated with serious intracranial vascular disorders, particularly subarachnoid hemorrhage; therefore, it is necessary to exclude this and a series of other pathological conditions, including intracerebral hemorrhage, cerebral venous thrombosis, vascular malformations (mainly aneurysm), arterial dissection (intra- and extracranial), reversible cerebral vasoconstriction syndrome (RCVS), pituitary apoplexy and, secondarily, infectious diseases and disorders due to altered cerebrospinal fluid pressure [11]. Thus, the diagnosis of a primary form must be a diagnosis of exclusion with normal neuroimaging and cerebrospinal fluid examination.

### 3.1. Epidemiology

Evidence and published data on secondary thunderclap headaches in children, compared to adults, remain limited. The only study conducted in children and adolescents aged 6–18 years reports a prevalence of 0.8% [11]. In this retrospective study, only children aged 6–18 years were included because younger children might have difficulty comprehending conventional pain scales and might fail to report the sudden onset of the headache. This bias could have led to an underestimation of thunderclap headache prevalence in childhood. In this study [11], 52% of patients with thunderclap headache were female. The mean age was 14.13 ± 4.12 years (median 16 years). The mean attack duration was 9.1 ± 8.7 h (median 6 h). All patients diagnosed with thunderclap headache had a pain score of 10; moreover, all had a negative neurological examination, normal funduscopic exam, negative neuroimaging (CT and/or MRI), and a favorable course [11].

### 3.2. Primary Thunderclap Headache

As in adults, almost 50% of thunderclap headaches in childhood are classified as primary thunderclap headache [13]. In a retrospective study conducted in a pediatric emergency department over three years, nineteen children (0.08%) were diagnosed with thunderclap headaches. Fifteen (79%) patients with thunderclap headache were diagnosed with a primary headache: six (31.6%) with migraine, eight (42.1%) with primary thunderclap headache, and one with another type of headache. All had normal neuroimaging (CT and/or MRI) and a favorable course [11].

### 3.3. Secondary Thunderclap Headache

Intracranial vascular diseases—the main causes of secondary thunderclap headache in adults—are less common in children. During a 3-year study period [13], only two children aged 6–18 years had a headache secondary to hemorrhagic diseases: one cavernous hemangioma and one cerebral venous thrombosis, neither of whom presented with thunderclap headache. In the same study, of the nineteen children with thunderclap headache, only four (21%) had secondary thunderclap headache; of these, one child presented malignant hypertension, and the remaining three had infectious diseases. All nineteen children and adolescents with thunderclap headaches had a favorable course.

In a case report, Norbedo et al. [14] described a 16-year-old boy with a thunderclap headache secondary to subarachnoid hemorrhage. The boy had hypertension secondary to a secreting paraganglioma and was diagnosed with hereditary paraganglioma–pheochromocytoma syndrome. The incidence of hemorrhagic stroke in children is 1.1–1.4 per 100,000 children [15,16], much lower than in adults (51.4–57.3 per 100,000) [17].

In adults, neurovascular diseases are often related to diabetes, dyslipidemia, smoking, and atrial fibrillation, conditions that rarely affect children [18]. Therefore, the higher prevalence of primary thunderclap headaches in childhood may reflect the lower occurrence of severe neurovascular conditions in children compared to adults.

### 3.4. Diagnosis of Thunderclap Headache in Childhood

The diagnosis of thunderclap headache requires a systematic and rapid approach to identify and promptly treat potentially serious causes. It is essential to obtain a detailed history, investigating the family history of neurological or vascular disease, and to assess the characteristics of pain or any associated symptoms (autonomic symptoms and other systemic symptoms).

In this context, accurate diagnosis requires the use of reliable pain-assessment instruments. For children, using a scale validated for pediatric age becomes necessary. Subjective scales represent the gold standard for assessing pain in older children.

The Visual Analog Scale (VAS) and the Faces Pain Scale—Revised are the two most commonly used scales in this group. In the VAS, the child indicates pain intensity on a 10 cm line with two extremes: one corresponding to “no pain” and the other to “worst pain.” A score is obtained by measuring the distance (in mm) between “no pain” and the child’s mark. The Faces Pain Scale is another subjective scale in which faces express different degrees of distress. It is used mainly for younger children who may have difficulty with tools requiring greater cognitive skills, for children with intellectual disabilities, or for those who do not understand the VAS explanation. A strong correlation has been found between the two scales [19].

**Figure 1:**
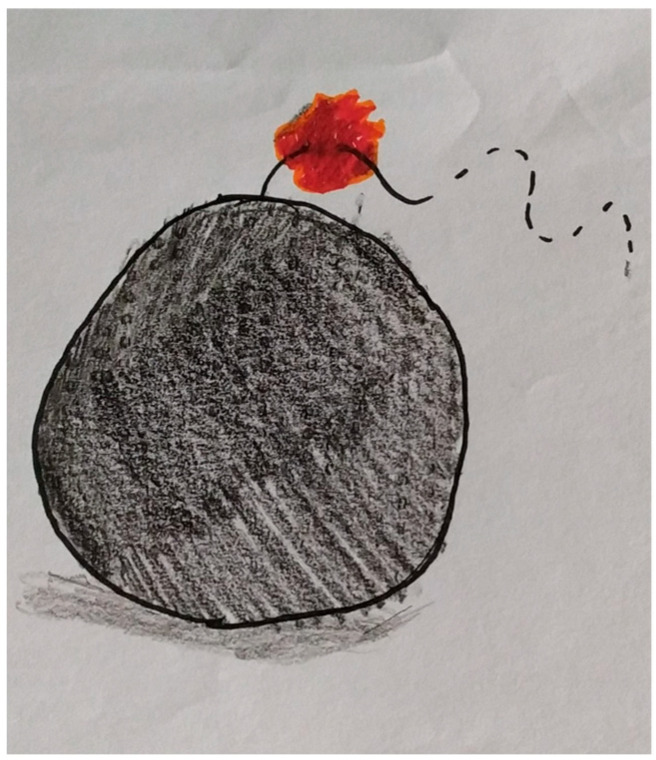
Thunderclap headache illustrated as explosive pain by a child. Cited from Ferro F. et al., 2025 [20].

The diagnostic work-up then includes a complete neurological examination, funduscopic examination, laboratory tests, brain CT and MRI, and lumbar puncture if the CT scan is negative but there is strong clinical suspicion. In Figure 1 we report the thunderclap headache illustrated by our child.

### 3.5. Management and Treatment

Management and treatment of thunderclap headaches are based on the underlying cause. Serious conditions such as subarachnoid hemorrhage, arterial dissection, or cerebral venous thrombosis require in-hospital monitoring. Pharmacological treatment is directed toward the underlying cause (e.g., anticoagulants for venous thrombosis, calcium-channel blockers for RCVS, antibiotics/antivirals for infections, and surgical or endovascular procedures for aneurysms, arteriovenous malformations, or arterial dissections).

Follow-up focuses on monitoring the response to treatment, detecting any complications, and providing psychological support to the patient and family to manage anxiety and understand the condition.

## 4. Cold-Stimulus Headache

Cold-stimulus headache (CSH) is a primary form of headache induced by contact and/or exposure of the head to a cold thermal stimulus, or by the ingestion and/or inhalation of cold substances (see Table 3) [1]. Although it is frequently observed in the general population, the pediatric scientific literature dedicated to this condition is surprisingly limited [21].

### 4.1. Epidemiology

From the available data, the prevalence of CSH in children is significantly higher than that observed in adults, with values ranging between 40.6% and 79% [22,23,24]. Unlike adulthood, no significant differences between males and females are seen in children. A strong association has also been reported between CSH and a personal history of migraine, as well as a clear correlation with a positive family history of the same condition [24].

### 4.2. Pathophysiology

In the pediatric population, the triggers described are generally related to the ingestion of cold substances (ice cream, cold water, ice cubes), whereas forms provoked by environmental cold have so far been rarely reported [21].

The main pathogenetic hypotheses include vasoactive phenomena (vasoconstriction followed by vasodilation), activation of nociceptors in the pharyngeal region, and possible involvement of the trigeminal–autonomic reflex [25,26]. In individuals with migraine, a segmental disinhibition of central pain circuits has been hypothesized [27].

### 4.3. Clinical Features

Pain is usually described as acute, throbbing, or stabbing, with rapid onset (within 10–60 s of the stimulus) and short duration (1–5 min). The location is predominantly frontal or temporal and often bilateral. In migraineurs, the area tends to coincide with that of their usual headache [28].

### 4.4. Treatment

There are no specific therapies because attacks are self-limited. The main strategy is prevention through gradual exposure to cold stimuli or avoidance of triggers [23].

### 4.5. Conclusions

In conclusion, CSH in children is more frequent than generally perceived and shows features that differ from adulthood. Further studies are required to better understand the pathophysiological mechanisms and to develop appropriate clinical guidelines [21].

## 5. Primary Stabbing Headache

Primary stabbing headache (PSH), according to ICHD-3 [1], is an epicranial headache characterized by well-localized, stabbing pain of short duration, like jabs or electric shocks, that appear suddenly as single stabs or in clusters, in the absence of other organic diseases of the anatomical structures or cranial nerves. The duration usually does not exceed 3 s, rarely reaching 10–20 s, with a low daily frequency and a variable site in two-thirds of cases. Accompanying symptoms occur in a minority of patients, excluding cranial autonomic symptoms (CAS). In patients with migraine, primary stabbing headache occurs ipsilateral to the migraine pain [1].

Initially classified as “idiopathic stabbing headache,” it became “primary stabbing headache” in 2004, whose diagnostic criteria included distribution of pain in the first trigeminal division, absence of accompanying symptoms, and a positive response to indomethacin [1]. In ICDH-3 [1], as seen in Table 4, the diagnostic criteria were revised to no longer include pain localization (which in 70% of cases may involve extratrigeminal regions) and, among accompanying symptoms, only CAS are excluded. Additionally, the ICHD-3 defines ‘probable primary stabbing headache,’ which requires only two of the criteria B–D to be fulfilled (Table 5). This classification is particularly relevant when the duration criterion is not satisfied. This entity is widely studied in adults, but there are still few pediatric studies and limited data, despite its relatively high prevalence in children. Moreover, some features in pediatric forms differ from adult manifestations and thus from ICHD-3 criteria.

A recent 2024 review [29] analyzed 12 studies (1991–2023) with the aim of evaluating the epidemiology, clinical manifestations, and treatment of primary stabbing headache and probable primary stabbing headache (according to ICHD-3) in pediatric patients (0–18 years), summarizing the main data in the literature.

### 5.1. Epidemiology

From the few pediatric studies available, primary stabbing headache and probable primary stabbing headache show a prevalence ranging between 2.5% and 10% among children with primary headache, with a higher percentage under 6 years of age and an age at onset between 7 and 11 years [29]. In a recent 2024 retrospective study, 60 patients attending headache centers in Rome and Bari between 2016 and 2022 were enrolled, with a mean age at onset of 8 years [30]. 

Several studies found no sex-related prevalence differences, although one study [31] showed a higher prevalence in females with an F:M ratio of 2:1, mirroring adult studies.

### 5.2. Clinical Features

In childhood, clinical characteristics can differ from adults; attack duration, for instance, ranges from a few seconds to several minutes (10–15) [32,33] and pain intensity from moderate to severe. A recent study reported that the duration of the stabs varies from a few seconds to up to 30 min, occurring with irregular frequency [30].

In a 2019 study of 42 patients, up to 50 stabs could occur in each attack, with frequency ranging from daily to monthly [34].

Associated symptoms such as photophobia, dizziness, nausea, and vomiting are rare. Neurological examinations are essentially normal, and the EEG may show sporadic epileptiform abnormalities in up to 30% of cases. Pain is usually unilateral, mostly anterior, although a possible occipital location has been described (more frequently in adults).

A positive family history of headache is often present (31–58% of patients), especially for migraine [29]. A summary of the differences in clinical features between children and adults is provided in Table 6.

### 5.3. Pathophysiology

Primary stabbing headache is often associated with other primary headaches, especially migraine and periodic syndromes (47% [34]) suggesting a common pathophysiological substrate related to central and peripheral sensitization mechanisms. Nevertheless, in the pediatric population, the association with other primary headaches is lower than in adults [31,33,35].

The high prevalence in young children may also suggest the possibility of an early phenotypic variant of migraine [31].

### 5.4. Treatment

Studies show conflicting results on the actual effectiveness of preventive therapy, which currently relies on indomethacin, trazodone, valproate, and amitriptyline [29], and is reserved only for certain categories of patients, depending on attack frequency and severity, and any association with migraine.

According to a 2020 study, prophylaxis with indomethacin at a dose of 25 mg three times daily may be successful in patients with many daily stabs and/or pain that interferes with normal school, family, and social activities, with the option to discontinue treatment 6–8 weeks after remission [36].

Indeed, primary stabbing headache belongs to the group of headache disorders known as “indomethacin-responsive headaches,” i.e., disorders characterized by partial or complete response to indomethacin. This category includes paroxysmal hemicrania, hemicrania continua, cough and exertional headaches, headache associated with sexual activity, primary stabbing headache, and hypnic headache [37].

A 2022 study analyzed indomethacin-responsive headaches in children and adolescents, enrolling 32 pediatric patients (16 females) who had at least 80% improvement with indomethacin [38]. Of these 32 patients, 13 had hemicrania continua, 1 paroxysmal hemicrania, 2 primary stabbing headache, 1 SUNCT (short-lasting unilateral neuralgiform headache attacks with conjunctival injection and tearing), 1 primary exertional headache, and 1 primary cough headache, with a mean age at onset of 10.9 years (range 2–16). In patients weighing >45 kg, the mean initial dose was 56 mg/day (range 12.5–75) and the mean final dose 122.3 mg/day (range 25–300). In patients weighing <45 kg, the mean initial dose was 1.1 mg/kg/day (range 0.2–18) and the mean final dose 2.5 mg/kg/day (range 1.1–5.4). Response to indomethacin was complete (100% pain resolution) in 24/32 patients; 2 improved by 95%, 2 by 90%, 1 by 85%, and 3 by 80%. Adverse events occurred in 41% of patients, mainly gastrointestinal symptoms, which improved or were eliminated by combining with gastroprotective agents [38].

### 5.5. Conclusions

This primary headache, therefore, shows characteristics that differ in the pediatric population compared with adults (Table 6): duration is longer in children, reaching even 15 s (rather than 3). Although an association with other primary headaches exists, it is less frequent than in adults [31,33,35].

**Figure 2:**
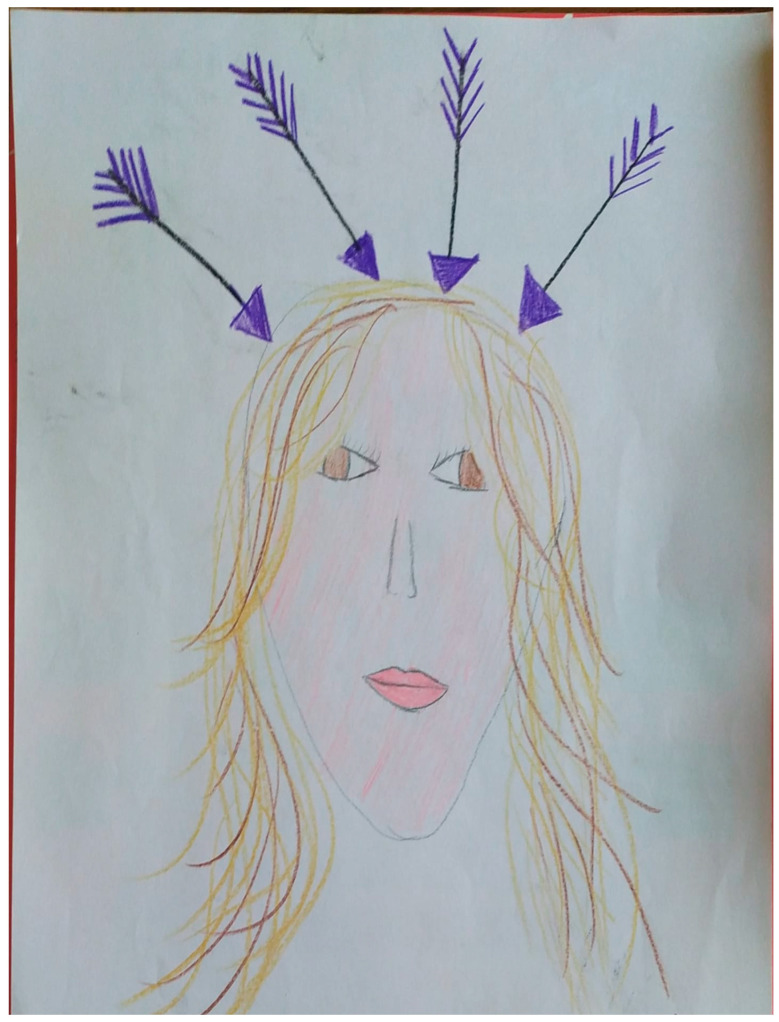
Primary stabbing headache illustrated by a 13-year-9-month-old girl. Cited from Ferro F. et al., 2025 [20].

In addition, in children, it is more difficult to distinguish a single longer stab from a cluster of stabs, and drawing can be a useful tool for describing the pain. Figure 2 shows the drawing of an 8-year-old girl illustrating her headache, characterized by stabs like “sword blows” to the head.

For these reasons, primary stabbing headache—despite its high prevalence in the pediatric population—is often underdiagnosed, which is why, as highlighted in several studies [30,34,36], a redefinition of the ICHD-3 diagnostic criteria should be warranted to make them more suitable for pediatric diagnosis. In Figure 2 we report the primary stabbing headache illustrated by our child.

## 6. Pediatric Nummular Headaches

The Nummular Headache (NH), previously named Coin-shaped headache [39], is a rare primary headache whose incidence in adults was estimated at around 6.4/100,000/year [40,41]. It is characterized by pain localized in a small, circumscribed area of the scalp, typically ranging from 1 to 6 cm in diameter. The pain is frequently localized in the parietal region, sharply contoured, fixed in size, with a round or elliptical shape. It is usually of moderate intensity, though it can occasionally be severe, and its duration is variable: it may be continuous or intermittent (ranging from seconds to days), or chronic (longer than three months) [1].

The exacerbations may manifest as lancinating pain lasting only a few seconds or may build up gradually over 10 min to 2 h, superimposing the baseline pain. The long-lasting exacerbations are more frequent than the ultra-brief ones, with minor fluctuation in their baseline and with chronic or remitting temporal patterns. Most patients (70%) report a gradual onset of pain, while the remaining (30%) experience a sudden onset [42]. The affected area may present hypoaesthesia, dysesthesia, paresthesia, allodynia, and/or tenderness. Additional symptoms, such as phonophobia, photophobia, and autonomic symptoms, are rarely reported, regardless of whether a coexisting headache is present [42].

It is uncertain whether NH represents neuralgia of a terminal branch of a pericranial sensitive nerve, or it is a focal nociceptive type pain derived from epicranial tissues [43].

The topography of the painful area and the associated sensory symptoms support the peripheral cause. However, evidence also points toward a central origin: subcutaneous injections of anesthetics were often unsatisfactory, and some patients described a painful area that extended across the midline of the scalp, or a bifocal/multifocal headache [42,43]. Therefore, it cannot be ruled out that the pathogenesis of NH may involve both central and peripheral mechanisms.

### 6.1. Epidemiology

According to the literature, NH is very rare in the pediatric population [44]. Incidence and prevalence are low, suggesting that NH is less common and/or underdiagnosed in children; a study of primary headache conducted in a population of 2466 children in Korea described a prevalence of 0.3% [45]. No significant differences between males and females are reported, although a case series described a female predominance of around 64.4%. [46].

### 6.2. Diagnosis

The diagnosis of pediatric NH is probably underestimated. Indeed, in one case, the pain started at the age of 6, and NH was diagnosed at age 41 [46]. In another case, the diagnosis was made ten years after the onset of pain [46]. The MRI and CT scan are usually normal; however, there are reported cases in which underlying lesions, such as meningioma and arachnoid cysts, have been implicated in the development of secondary forms of NH [44]. The neurologic examination and the blood examinations are also normal in NH patients. In Table 7, we reported the ICHD-3 diagnostic criteria for nummular headache [1].

### 6.3. Treatment

Treatment of NH in the pediatric population remains difficult. In cluster attacks, it can be used as a symptomatic therapy (e.g., ibuprofen) [44,46]. In many cases, treatment for comorbid migraine (especially topiramate), combined with lifestyle modification and vitamin supplements (coenzyme Q10, vitamin D, folate), when a deficiency is detected, may be effective [44]. Related to this topic, Panda et al. [47] reported a case of an 11-year-old girl diagnosed with NH. The headache started with moderate intensity and gradually increased in severity, becoming debilitating. The pain did not respond sufficiently to non-steroidal anti-inflammatory drugs (NSAIDs) and other medications for migraine (flunarizine, topiramate, propranolol, amitriptyline). The patient partially responded to carbamazepine (titrated to 30 mg/kg/day) and was headache-free when she started an add-on therapy with gabapentin (600 mg/day).

There are pediatric cases of new daily persistent headaches with the NH phenotype, refractory to several therapies (valproate, amitriptyline, cyproheptadine, topiramate, vitamin D, coenzyme Q10, and occipital nerve block with naproxen). In severe cases, it is also described as a beneficial effect of the Onabotulinum toxin A (BoNT-A) [46].^.^

In secondary cases such as the one associated with Langerhans cell histiocytosis, lesionectomy proved to be beneficial, although not all the secondary cases responded to surgery [44].

### 6.4. Open Questions—Physiopathological Hypothesis

**Figure 3:**
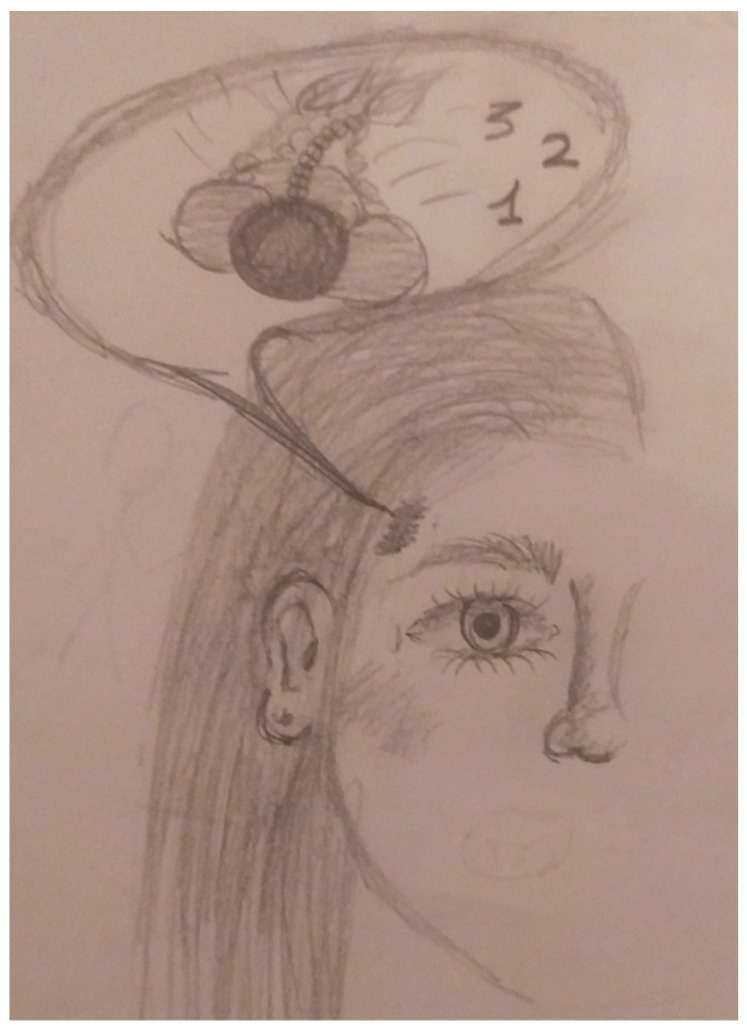
Drawing of a 14-year-old girl complaining of pulsating pain in a circumscribed rectangular area. The numbers drawn represent the severity grading that the headache can reach.

We recently reported six new cases of pediatric nummular headache, two of which reported involvement of a rectangular, not circular, area, exhibiting the pattern of the affected area (see Figure 3) [44]. 

For this reason, we asked ourselves what pathophysiological basis underlies the circularity of the lesion that characterizes the syndrome.

The main critique is that the definition relies on a circular/oval shape for the pain area (which is a mandatory criterion), yet there is no clear scientific explanation for why the pathogenesis (peripheral or central) should produce that specific shape.

A more logical diagnostic criterion, in our opinion, should simply be a fixed and restricted pain area in size and location, and the circular shape should be considered only one of the possible findings, not an essential requirement [44].

So why in nummular headache (NH) is the pain area almost always described as perfectly circular or oval, even though neuroanatomical evidence suggests it should be irregular?

Current studies on the spatial resolution of pain do not provide clear answers, as research specifically on the scalp is lacking [48,49]. However, we know that the abundance of hair and the fragmented nature of nerve fiber receptive fields should, in theory, generate an irregular and poorly defined pain perception. Since the pain perceived by the patient is, conversely, regular and well-defined, this discrepancy indicates that peripheral mechanisms alone are insufficient to explain the phenomenon.

We recently proposed a hypothesis to shift the focus to the center [50], invoking the active inference [51]. According to this model, perception is not a passive process but rather an inference: the brain constructs sensory experience based on prior expectations and internal predictive models (top-down processing). The stable, regular shape of NH could therefore not reflect direct nociceptive input, but rather the imposition of an internal predictive model. Essentially, the brain decides and actively constructs that precise spatial representation of the pain.

A further hypothesis strengthens this framework by linking perception to motion. When localized pain is felt, people naturally tend to touch or massage the area using curvilinear or circular movements. The brain may incorporate this repeated motor action as a sensory expectation (prior). This continuous feedback, combining nociception with circular movement, leads the brain to infer and therefore construct the painful area’s shape as circular [50].

Finally, a more methodological possibility is raised: selection bias by researchers. By concentrating on the mandatory circular/oval shape criterion to diagnose this rare syndrome, they might inadvertently overlook or underestimate the existence of other possible pain shapes [44]. For these reasons, we suggested modifying the ICHD-3 diagnostic criteria [44].

However, our hypotheses require experimental validation through extensive clinical studies of subjects with localized headaches, whose areas are defined using instruments such as pressure algometers or experimental animal models.

### 6.5. Conclusions

Nummular headache certainly requires further physiopathological and nosographic investigation to better define and diagnose it.

## 7. Hypnic Headache

According to the third edition of the International Classification of Headache Disorders (ICHD-3) [1], hypnic headache (HH) is defined as a primary headache characterized by recurrent attacks that occur exclusively during sleep, causing the patient to awaken. Diagnosis requires attacks to occur ≥10 times per month for over three months, lasting 15 min to 4 h after awakening, without cranial autonomic symptoms (such as tearing, rhinorrhea, or ptosis) or psychomotor agitation. In addition, the symptoms must not be better accounted for by another ICHD-3 diagnosis. The Table 8 reports the diagnostic criteria ICHD-3 [1]

First described by Raskin in 1988 [52], it is commonly observed in the elderly but may occasionally occur in childhood, although extremely rarely [53].

Neurophysiological and neuroradiological investigations (brain MRI, EEG, blood tests), when performed, do not show significant abnormalities, confirming the primary nature of the headache [53].

Hypnic headaches seem to be very rare in the pediatric population. A systematic review aimed to comprehensively describe the clinical characteristics of published cases of primary hypnic headache in children, as the condition’s underlying mechanisms are not fully understood, showed the necessity of developing diagnostic criteria specific to pediatric cases to improve diagnosis rates and the overall management of children suffering from HH [54].

The review involved a systematic search across major medical databases covering the period from 1988 to 2023 to identify articles reporting HH in patients under 18 years of age.

The results showed a mean age of onset around 10 ± 4,3 years (range 3–15 years), with a mean time from onset to diagnosis of 15.8 ± 25.0 months (range 1–60 months). The findings also indicated that headache characteristics in children differed significantly from those of adults: children typically experienced pulsating pain, while adults reported dull/pressing pain. Additionally, children presented with a lower frequency and shorter duration of attacks. 

### 7.1. Physio Pathological Hypothesis

Pathogenetic hypotheses suggest a disturbance of the circadian rhythm, likely related to dysfunction of the suprachiasmatic nuclei of the hypothalamus and dysregulation of melatonin secretion [55,56,57]. The higher incidence of HH in adults might be explained by hypothalamic dysfunction and melatonin dysregulation, which are more prevalent in older individuals. However, the pathophysiological mechanisms in children remain poorly understood [53].

### 7.2. Treatment

Acute treatment is generally unnecessary due to the limited duration of attacks. In two cases reported in the literature, administration of melatonin (2–4 mg) led to a significant reduction in the frequency and intensity of headache, up to complete remission [54].

### 7.3. Conclusions

In conclusion, hypnic headaches in childhood are an exceptional but real condition that is often underrecognized. Early identification can avoid invasive diagnostic workups and allow effective management with targeted treatments.

## 8. Red Ear Syndrome

Red Ear Syndrome (RES) is a fairly rare disorder first described by Lance in 1996 [2]. It is characterized by the gradual or sudden onset of unilateral or bilateral redness of the ears with an annoying sensation of heat, burning, and the eventual presence of edema and painful sensation evoked by palpation of the ear. The attacks can last from minutes to several hours, but usually last less than an hour, and can be very frequent. Three forms can be identified: an idiopathic form, in which the red ear occurs in isolation; a secondary form, determined by external causes; and a form linked to primary headaches, which may manifest in both adults and children. In older individuals, symptomatic forms are more common, whereas in younger patients, idiopathic or primary-headache–associated forms predominate [58].

### 8.1. Epidemiology

It is described as a rare form of facial pain; however, over 100 cases have been described. However, in a study involving young individuals, it has been observed in approximately 20% of subjects with primary headaches accompanied by other parasympathetic cranial autonomic symptoms. Numerous cases have been described in pediatric age, including a case in a 3-year-old child [59].

Probably, as reported by Boulton et al. [60], it can be underestimated due to its transitory temporal course and a lack of knowledge; thus, the diagnostic suspicion tends to arise when the frequency of attacks is very high and becomes unbearable.

### 8.2. Pathophysiology and Clinical Features

From a physiopathogenetic standpoint, the most accepted hypothesis involves autonomic parasympathetic activation, particularly in forms associated with primary headaches and probably also for the idiopathic form, despite the parasympathetic innervation of the ear being relatively limited compared with the sympathetic component. Alternative mechanisms, such as an axonal reflex or a localized sympathetic deficit at the ear, have also been suggested. Indeed, sensory innervation is provided by the auriculotemporal nerve of the third branch of the trigeminal nerve in the anterior part and by the cervical branches in the posterior part of the ear. The auricolar region receives a vascular supply from the branches of the middle temporal and posterior temporal arteries (external carotid circulation, innervated by the trigeminal nerve). Vasomotor tone exerts mainly a vasoconstrictor effect through sympathetic control (RES vasodilation could be due to inhibition of sympathetic fibers), while parasympathetic vasodilatory control is marginal, mainly related to the forehead and cheeks (this could explain RES associated with facial trigeminal autonomic cephalalgias (TACs) or migraines) [58,61].

Primary headaches can be triggered by numerous stimuli such as local or general heat in the external environment, rubbing or touching the ear, chewing, grinding, stress, dietary factors, etc. These subjects frequently have a history of primary headache, particularly migraine (about 75%), but the attacks occur in isolation and are separate from the headache attacks [58].

Forms associated with primary headaches have been described mainly during migraine attacks, but also during cluster headaches and other TACs [57,59]. Other autonomic signs, such as tearing or facial flushing, are often associated. Mechanical pain may precede the attack or be present simultaneously. The affected ear is typically ipsilateral to the pain, but bilateral involvement may occur [58].

### 8.3. Diagnosis

**Figure 4:**
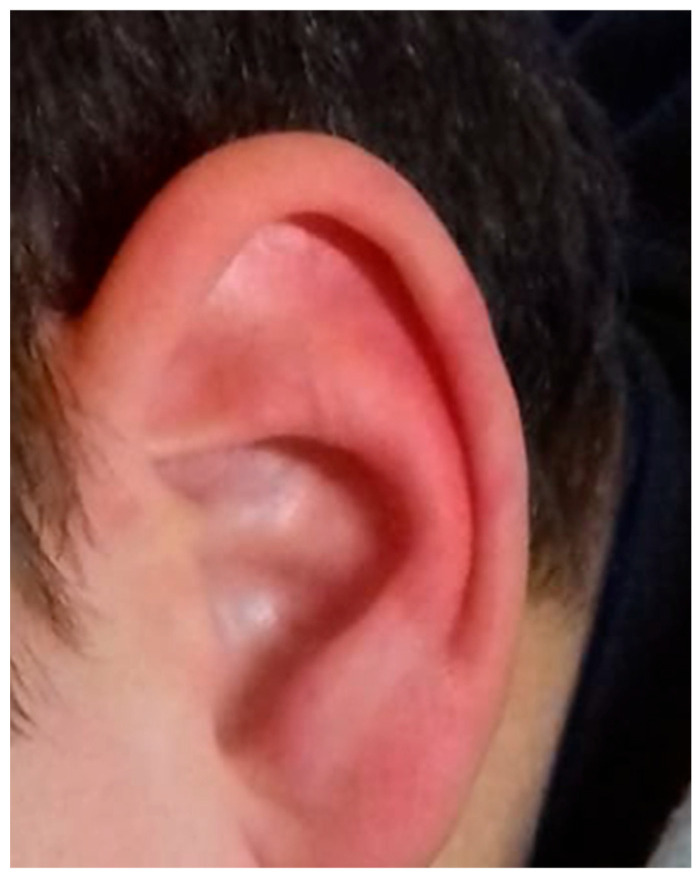
Child with Red Ear Syndrome. Photo from D’Amico et al. 2021 [59].

The diagnosis can be straightforward due to its clearly visible characteristics (Figure 4) if the physician is aware of its existence, while the differential diagnosis must be made primarily by excluding secondary causes, erythromelalgia, and urticarial forms in the facial region.

Secondary forms are due to deficits in the sympathetic system due to compression of the nerve bundle at the cervical level, Arnold-Chiari malformation type 1, temporomandibular joint disorder, neurovascular compression, or from drugs or ischemic thalamic lesions [58].

### 8.4. Treatment

Published cases of RES are characterized by their particular resistance to pharmacological treatments, which is often extremely bothersome, and in many cases, various types of drugs, including NSAIDs, indomethacin, beta blockers, calcium antagonists, tricyclics, and antiepileptics, have been used with variable success. Treatments with botulinum toxin, high doses of steroids, local infiltrations of lidocaine, ice compression, nerve blocks, or ultrasound treatments have also been reported. This multiplicity of treatment attempts demonstrates that, although uncommon, RES is extremely bothersome in its more invasive forms, and its treatment remains extremely challenging [58].

Raieli et al. [58] report a complete table of the various treatments reported in the literature up to 2016, also citing the number of cases and therapeutic responses. D’Amico et al. [59] report a similar table referring only to pediatric cases cited in the literature up to 2021.

In 2013, Lambru and collaborators [61] suggested to insert the Idiopathic RES in Group IV of the International Classification of Headache Disorders [1], reporting possible diagnostic criteria for the diagnosis of red ear syndrome (see Table 9), but in several pediatric cases, some aspects, such as unilaterality, are not applicable. However, even now, red ear syndrome is not recognized by the current IHS international classification of headache disorders, compared to rarer forms such as external compression headache or primary trochlear headache [62].

## 9. Notes on Non-Dental Orofacial Pain in Childhood

Orofacial pain syndromes (OFPs) are a heterogeneous group of pain syndromes characterized by painful attacks involving structures of the craniofacial district “below the orbitomeatal line, anterior to the auricle, and above the neck,” and they often entail a complex diagnostic–therapeutic journey. Recently, in an attempt to bring order to the numerous pain syndromes and establish precise diagnostic criteria, a consensus among experts from various international societies (e.g., the International Headache Society, the International Association for the Study of Pain, etc.) produced the first International Classification of Orofacial Pain (ICOP) [3].

According to the ICOP classification, orofacial pain syndromes can be divided into six groups:

1. OFP is attributed to disorders of the dento-alveolar structures and related anatomy.
2. Orofacial myofascial pain.
3. Temporomandibular joint (TMJ) pain.
4. OFP attributed to a lesion or disease of the cranial nerves.
5. OFP with features overlapping primary headaches.
6. Idiopathic OFP.

Syndromes due to odontogenic causes, myofascial disorders, or temporomandibular joint disorders are very common at different ages, whereas the remaining groups may be underdiagnosed and inappropriately treated, including invasive dental procedures [63].

The last three groups are very rare during childhood and adolescence and may underlie secondary causes [64,65]; therefore, it is important to define clinical characteristics appropriately and to maintain a high level of suspicion during the diagnostic process. Forms belonging to groups 4–5 and 6—including typical and atypical neuralgias, “burning mouth” syndrome, and trochlear migraine—are uncommon, especially in childhood, and they risk being underestimated and possibly confused with dentoalveolar or temporomandibular disorders, and thus represent a real challenge for the clinician planning the diagnostic and therapeutic path [66]. It is possible to distinguish typical and atypical forms based on characteristics such as duration and location of the pain—which may be musculoskeletal, neurovascular, or neuropathic—and clinical expression [64,65].

Neuralgic conditions—including trigeminal neuralgia and neuralgia of the glossopharyngeal, intermediate, and occipital nerves—are very rare and disabling forms of facial pain that present as unilateral or bilateral lancinating paroxysms and may underlie secondary causes that must always be carefully investigated in childhood. Finally, there are atypical facial pains characterized by idiopathic persistent pain—deep in the soft tissues or bone—burning or severe and throbbing, that does not fulfill any other diagnostic criteria; among these is “burning mouth syndrome” (BMS), characterized by a dysesthetic intraoral sensation confined to the mouth or tongue [64].

### 9.1. Case Series

**Figure 5:**
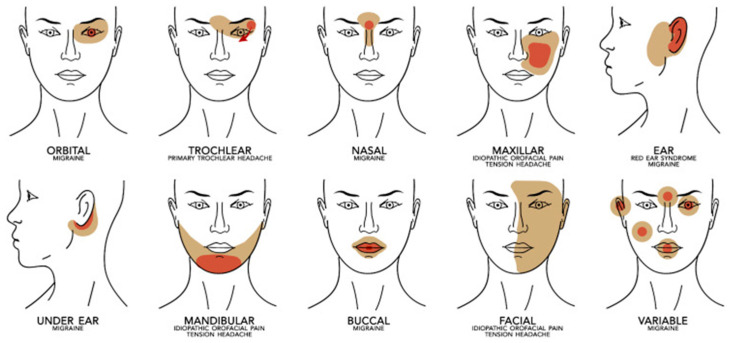
Distribution of pain in the anatomic area with prevalent associated diagnosis. Red represents the focal point of pain presentation, and mustard represents the adjacent areas of prevalent irradiation. From Correnti et al. 2023 [67].

Through collaboration among three Italian headache centers (Bari, Palermo, Turin), it was possible to collect a series of pediatric clinical cases extracted from data gathered between 2017 and 2021 [67], sharing the feature of non-dental facial pain according to the topographic criteria of the 3rd International Classification of Headache Disorders (ICHD-3). The sample consisted of 43 subjects (23/20 M/F; age 5–17 years), subdivided as follows: 23 primary headaches involving the facial territory during attacks, 2 facial trigeminal autonomic cephalalgias (TACs), 1 primary facial stabbing headache, 1 facial linear headache, 6 trochlear migraines, 1 orbital migraine, 3 RES, and 6 atypical facial pains. All patients described disabling pain in terms of intensity (moderate/severe); 31 children had episodic attacks, and 12 had continuous pain. Figure 5 shows the main locations of the various pain syndromes. 

Almost all of the subjects received medications for acute treatment (fewer than 50% were satisfied), and some received non-pharmacological treatment in addition to drug therapy.

From this work, the following conclusions can be drawn: although OFPs are rare in childhood, they can be very debilitating if not recognized and adequately treated, affecting the psychophysical well-being of young patients. Moreover, primary headaches may be indistinguishable in clinical characteristics from classic syndromes; however, due to their atypical location, they may escape attention and be confused with orofacial pain due to dental causes or secondary to sinus inflammatory processes.

### 9.2. Conclusions

A multidisciplinary approach to these conditions is certainly the best option (pharmacological and non-pharmacological treatment plus psychological counseling) to reduce the intensity and frequency of pain and to reduce the negative impact on quality of life and prevent possible disability in adulthood.

## 10. Conclusions

In summary, this review underscores the critical need for increased awareness regarding rare primary headache disorders, including cough, thunderclap, cold, primary stabbing, nummular, and hypnic headaches, as well as Red Ear Syndrome and non-odontogenic orofacial pain.

The presented overview of the clinical, therapeutic, and pathophysiological aspects of these conditions highlights that their infrequent occurrence often leads to diagnostic delay and subsequent inadequate management. Addressing these complex clinical entities is crucial, as they currently represent a significant diagnostic challenge for practitioners and a considerable source of serious disability for children and adolescents.

Given the rarity of these disorders in children, it is always important to perform a diagnostic work-up to avoid overlooking possible secondary causes. Table 10 lists the main red flags, possible secondary causes for these rare primary headaches, and the main diagnostic tests indicated.

Crucially, the current diagnostic criteria may not fully capture the clinical spectrum of these rare forms in the developing pediatric population. Therefore, future research must focus on revising and adapting the existing diagnostic criteria to better reflect the specific manifestations and characteristics observed in children and adolescents. This essential step, alongside educational initiatives, is vital to improve diagnostic accuracy and ensure optimal therapeutic outcomes for these specific, often overlooked, pediatric headache forms.

## Figures and Tables

**Table 1:** Diagnostic criteria for primary cough headache (ICHD-3, Section 4.1) [1].

Primary Cough Headache
Diagnostic criteria: At least two headache episodes fulfilling criteria B-D. Triggered by and occurring only in association with coughing, physical exertion, and/or another Valsalva maneuver. Abrupt onset. Duration between 1 s and 2 h. Not better accounted for by another ICHD-3 diagnosis.

**Table 2:** Diagnostic criteria for thunderclap headache (ICHD-3, Section 4.4) [1].

Primary “Thunderclap” (or “Out of the Blue”) Headache
Diagnostic criteria: Severe headache fulfilling criteria B and C. Abrupt onset with peak intensity reached in <1 min ^1^. Duration > 5 min ^2.^ Not better accounted for by another ICHD-3 diagnosis.

^1^ Children and their parents usually do not report that the pain reaches maximum intensity in less than 1 min, but they report a severe headache with a sudden or abrupt onset. In adult studies [11,12], it is common practice to accept an abrupt onset as a satisfactory criterion. The use of this criterion is therefore suggested in children as well [13]. ^2^ If the child is unable to report the duration of the pain, this can be reported by parents.

**Table 3:** Diagnostic criteria for headache attributed to external application of a cold stimulus (ICHD-3, Section 4.5) [1].

Primary Cold-Stimulus Headache
Diagnostic criteria: ≥2 episodes of acute headache fulfilling B and C. Triggered by a cold stimulus applied to the head. Resolves within 30 min after removal of the stimulus. Not better accounted for by another ICHD-3 diagnosis.

**Table 4:** Diagnostic criteria for primary stabbing headache (ICHD-3, Section 4.7) [1].

Primary Stabbing Headache
Diagnostic criteria: Head pain that occurs spontaneously as a single stab or a series of stabs and fulfills criteria B-D. Each stab lasts no more than a few seconds ^1^. The stabs occur with irregular frequency, from one to multiple times per day ^2^. No cranial autonomic symptoms are present. Not better accounted for by another ICHD-3 diagnosis.

^1^ In 80% of cases, stabs last up to 3 s; they rarely reach 10–120 s. ^2^ Attack frequency is generally low (one or a few attacks per day). In rare cases, stabs recur for days; a status lasting one week has been described.

**Table 5:** Diagnostic criteria for probable primary stabbing headache (ICHD-3, Section 4.7.1) [1].

Probable Primary Stabbing Headache
Diagnostic criteria: Head pain that occurs spontaneously as a single stab or a series of stabs that fulfills only B-D criteria. Each stab lasts no more than a few seconds. The stabs occur with irregular frequency, from one to multiple times per day. No cranial autonomic symptoms are present. Does not fulfill ICHD-3 criteria for any other headache type. Not better accounted for by another ICHD-3 diagnosis.

**Table 6:** Differences and similarities in the clinical phenotype of PSH in children and adults.

Pain	Adults	Pediatric Population
Quality	Stabbing	Stabbing
Intensity	Moderate/Severe	Moderate/Severe
Location	Unilateral > Occipital	Unilateral > Frontal
Duration	≤3 s (rarely 10–120 s)	Up to 15 min
Accompanying symptoms	Infrequent	Infrequent
Association with other primary headaches	High association with migraine	Lower association

**Table 7:** Diagnostic criteria for nummular headache (ICHD-3, Section 4.8) [1].

Nummular Headache
Diagnostic criteria: Continuous or intermittent head pain fulfilling criterion B. Felt exclusively in an area of the scalp, with all of the following four characteristics: Sharply contoured; Fixed in size and shape; Round or elliptical; 1–6 cm in diameter. Not better accounted for by another ICHD-3 diagnosis.

Note: Other causes, in particular structural and dermatological lesions, have been excluded by history, physical examination, and appropriate investigations.

**Table 8:** Diagnostic criteria for hypnic headache (ICHD-3, Section 4.9) [1].

Hypnic Headache
Diagnostic criteria: Recurrent headache fulfilling criteria B-E. Occurs exclusively during sleep and causes the subject to awaken. Occurs on ≥10 days per month for more than 3 months. Lasts ≥15 min and up to 4 h after awakening. No cranial autonomic symptoms or motor restlessness are present. Not better accounted for by another ICHD-3diagnosis.

**Table 9:** Proposed diagnostic criteria for red ear syndrome [61].

Red Ear Syndrome
Diagnostic criteria: At least 20 attacks fulfilling criteria B-E. Episodes of external ear pain lasting up to 4 h. The ear pain has at least two of the following characteristics: Burning quality. Unilateral location. Mild to moderate severity. Stimulated by cutaneous or thermal stimulation of the ear. The ear pain is accompanied by ipsilateral redness of the external ear. Attacks occur at a frequency of ≥1 per day, although less frequent attacks may occur. Not attributable to another disorder.

**Table 10:** Red flags and differential diagnosis for pediatric primary rare headaches.

Pediatric Primary Rare Headaches	Main Red Flags for Secondary Headaches	Main Differential Diagnosis	Recommended Investigations
Cough Headache	Positive Valsalva Manoeuvre, Papilledema, Cough Headache	Arold Chiari Tipo 1 Brain Tumor, Hypertension Endocranial	Brain MRI
Thunderclap Headache	Abrupt Onset of Severe Pain	Cerebrovascular Diseases (Avm *, Sh **, Cvt ***) #	Brain CT, Brain MRI, Lumbar Puncture, Blood Pressure
Cold Stimulus Headache	Longer Duration, Different Location, Associated Neurologic or Cardiac Symptoms,	# Cardiac Cephalgia, Carotid, or Vertebral Dissection	Cardiologic investigations. Brain and Cervical MRI
Stabbing Headache	Longer Duration, Fixed Localization, Extracephalic Localization, Substantial Background Pain, Cranial Autonomic Symptoms, Local Allodynia and Dysesthesia	Tacs °, Trigeminal Neuralgia, Occipital Neuralgia, Pituitary Tumors, Idiopathic Hypertension Intracranial Pediatric Strokes, Multiple Sclerosis	Brain and Cervical MRI
Nummular Headache	Continuous Temporal Course, Local Hypoesthesia	Meningioma, Arachnoid Cysts, Langerhans Cell Histiocytosis	Brain MRI, Cranial Bone TC. Soft Tissue Ultrasound Scan of The Head
Hypnic Headache	Recent Onset of Pain, Continuous Time Course, Daytime Persistence of Pain Attacks, Vomiting	# Brain Tumors, Vascular Pathologies, Nocturnal Hypoglycemia, Nocturnal Hypertension, Medication Overuse, Osas °°	Brain MRI, Blood Pressure Monitoring, Metabolic Investigations
Red Ear	Recent Onset of Pain, Continuous Time Course, Fixed Unilaterality, Cervical Pain, Tinnitus, Skin Redness in Hands and Feet, Joint Pain, History of Autoimmune Diseases	Eritromelalgia, Cervical Disease, Arnold-Chiari Tipo 1, Lupus	Brain and Cervical MRI, Blood Investigations, Rheumatology Investigations, ENT Specialist Visit

Legend: * Arterovenous Malformation; ** Subarachnoid Hemorrhage; *** Cerebral Venous Thrombosis; # These Secondary Causes Have Been Described in Adults; There Are No Pediatric Descriptions; ° Trigeminal Autonomic Cephalalgias; °° Obstructive Sleep Apnea Syndrome.

## Data Availability

No new data were created.
